# Health Problems of Professional Ballet Dancers: an Analysis of 1627 Weekly Self-Reports on Injuries, Illnesses and Mental Health Problems During One Season

**DOI:** 10.1186/s40798-024-00753-1

**Published:** 2024-07-17

**Authors:** Astrid Junge, Anja Hauschild, Janine H. Stubbe, Rogier M. van Rijn

**Affiliations:** 1 Center for Health in Performing Arts and Institute of Interdisciplinary Exercise Science and Sports Medicine, Medical School Hamburg (MSH), Am Kaiserkai 1, 20457 Hamburg, Germany; 2 Center for Rehabilitation and Sports Medicine, BG Klinikum Hamburg, Hamburg, Germany; 3 Codarts Rotterdam, University of the Arts, Rotterdam, The Netherlands; 4 Performing Artist and Athlete Research Lab (PEARL), Rotterdam, The Netherlands

**Keywords:** Epidemiology, Prevalence, Musculoskeletal pain, Complaints, Performing artists

## Abstract

**Background:**

Several studies have investigated injuries of (pre-)professional ballet dancers, however most used a medical-attention and/or time-loss definition and did not analyse the prevalence of all health problems. The aim was to analyse the frequency and characteristics of all self-reported physical and mental health complaints (i.e. injuries, illnesses and mental health problems) of professional ballet dancers during one season.

**Methods:**

Three professional ballet companies were prospectively monitored weekly during one season with the Performing artist and Athlete Health Monitor (PAHM). Numerical rating scales (ranging 0–10) were used for severity of musculoskeletal pain, all health problems and impairment of the ability to dance at full potential in the previous seven days. If dancers rated the severity of their health problems or their impairment greater than 0, they were asked to answer specific questions on the characteristics of each health problem.

**Results:**

Over a period of 44 weeks, 57 dancers (57.9% female) filled in 1627 weekly reports (response rate of 64.9%), in which 1020 (62.7%) health problem were registered. The dancers reported musculoskeletal pain in 82.2% of the weeks. They felt that their ability to dance at their full potential was affected due to a health problem in about every second week (52.6%) or on at least 29.1% of the days documented in the weekly reports. Almost all dancers (96.5%) reported at least one injury, almost two thirds (64.9%) an illness and more than a quarter (28.1%) a mental health problem. On average, every dancer reported 5.6 health problems during the season. Most of the 320 health problems were injuries (73.1%), 16.9% illnesses and 10.0% mental health problems. Injuries affected mainly ankle, thigh, foot, and lower back and were mostly incurred during rehearsal (41.6%) or training (26.1%). The most frequent subjective reasons of injury were “too much workload” (35.3%), “tiredness/exhaustion” (*n* = 22.4%) and “stress/overload/insufficient regeneration” (*n* = 21.6%).

**Conclusion:**

Preventive interventions are urgently required to reduce the prevalence of health problems and especially injuries of professional dancers. Injury prevention measures should regard the balance of the load capacity of professional dancers and the workload in training, rehearsals and performances.

## Key Points

- The prevalence of health problems is high: Almost all professional ballet dancers (97%) reported at least one injury, 65% an illness and 28% a mental health problem during one season.
- Health problems have substantial impact: On at least 29% of the days documented in the weekly reports, the dancers were not able to dance at full potential due to a health problem.
- Many injuries seem to be preventable: Half of the injuries occur during training (26%) or rehearsal run-through (23%) and the most frequent reasons were “too much workload” (35%), “tiredness / exhaustion” (n=22%) and “stress / overload / insufficient regeneration” (22%).

## Background

Several studies have demonstrated the high incidence of injuries in (pre-)professional ballet dancers [1–7]. In almost all studies injuries were recorded by an in-house physiotherapist or the trainer and a medical-attention and/or time-loss definition was used. Whether or not an injury receives medical attention or results in time-loss can be influenced by several parameters, e.g. the availability of in-house medical staff, access to the public health care system, timely appointments, or pressure to perform. Jacobs et al. [3] found in a retrospective survey that more than 15% of all injured dancers had not reported their injury for various reasons. Thus, time-loss and medical-attention injury definitions underestimate the injury burden (e.g. [6, 8–10]). A further challenge is the recording of overuse injuries due to their mainly gradual onset and varying intensity over time [11]. Therefore, a research group at the Oslo Sports Trauma Research Centre (OSTRC) developed a self-report questionnaire for the registration of overuse injuries in sports injury epidemiology [12] and then extended the wording to cover all types of health problems [13].

The OSTRC questionnaire has been adapted for dancer populations [8, 14] and implemented in studies with pre-professional dancers [6, 15, 16]. A study among 130 contemporary dance and dance teacher students (mean age 19.4 years) showed that almost all students (96.9%) reported at least one health problem. A total of 620 health problems were reported of which 321 (51.8%) were injuries, 232 (37.4%) illnesses and 67 (10.8%) mental problems [15]. Another study on 452 pre-professional ballet dancers (mean age 15 years) reported a prevalence of 32.1% for time-loss injuries and 67.4% for all-complaint injuries across 5 years [6].

However, in a qualitative study on the user experience and content of the modified questionnaire professional dancers stated that the four OSTRC questions were unclear to them and were not applicable to their dance activities [14]. Further, all dancers had difficulties in classifying pain as an injury when they were still able to perform [14]. This observation is in agreement with Bolling et al. [17] who reported from a qualitative study on perception of injuries of professional dancers that “Participants defined an injury based mainly on dance performance limitations, while pain and time loss reflected injury severity. Dance injury was described as a spectrum of injury levels that depend on the ability to perform to the best of their ability, pain levels, and potential modification on dance participation.” [17]. Moreover, very few studies have assessed illnesses [15, 18] or self-reported mental health problems [15, 19] of dancers during the season, and no study on self-reported health problems included professional dancers. Therefore, the aim of the present study was to analyse the frequency and characteristics of self-reported injuries, illnesses and mental health problems in female and male professional ballet dancers.

## Methods

All dancers of the ballet companies of three German states theatres (*n* = 141) were asked to answer a baseline questionnaire at the start of the season 2021/22 and to report their health problems weekly using the Performing artist and Athlete Health Monitor (PAHM, [14, 15]) during the entire season.

The baseline questionnaire included questions on age, gender and rank (i.e. principal dancer, soloist, semi-soloist/coryphées/corps de ballet, eleve). The PAHM is a web-based system to pseudonymously record health problems (i.e. injuries, illnesses and mental health problems). The health record started with numerical rating scales (NRS) on severity of musculoskeletal pain, severity of all health problems (both NRS ranging from “not at all” (0) to “worst imaginable” (10)) and impairment of the ability to dance at full potential due to health problems (NRS ranging from “dance at full potential” (0) to “unable to dance” (10)) in the previous seven days. If the dancer rated the severity of their health problems or the impairment of their dance ability greater than “0” on the NRS, they were asked to classify their health problems as injury (defined as “musculoskeletal pain, complaints or injury, e.g. sore muscles, ankle sprain, concussion”), illness (defined as “illness or physical symptoms, e.g. influenza, diarrhoea, headache, menstrual pain”) or mental health problem (defined as “mental health issue, e.g. performance anxiety, depression”). Then they were asked to answer specific questions on the characteristics and consequences of each injury, illness or mental health problem, such as body part and cause of injury, kind of illness or mental health problem, consultation of a physician, physiotherapist, psychologist or another qualified medical practitioner, days unable to train, rehearse or perform due to the health problem and days the health problem affected their ability to dance at full potential. Categories for characteristics of injury (such as body areas, mode of onset, classification as new, recurrent or exacerbation) were based on the International Olympic Committee consensus statement: methods for recording and reporting of epidemiological data on injury and illness in sport [20] and adapted for the dancers (e.g. “worsening” were used instead of “exacerbation”, “new” was explained as “you never had this before”). The dancers were asked questions on the details of up to four health problems and to report further health problems as free text each week. Each health problem was only counted once when the dancers reported it for the first time. If an already reported health problem was reported again during the season, this was defined as a “follow-up report”. A health problem was classified as “time-loss” if the dancer regarded themselves as unable to dance due to this health problem on at least one day. Dancers were asked every Friday to fill in the PAHM, if they did not respond within 2 days, they received a reminder.

All dancers were informed about the content and aims of the study, and those participating gave written informed consent before the start of the study. The baseline questionnaire and the PAHM was pseudonymous (i.e. dancers used a personal code) to match the information. Only the individual dancer and two authors of the study (AH, RMvR) knew the match of code and name and kept this information strictly confidential and in accordance with the German data protection laws. The study has ethic approval (MSH 2021/137) of the Medical School Hamburg, Germany. The study was conducted in accordance with the Declaration of Helsinki.

The dancers were included in the analysis if they answered the baseline questionnaire and filled in at least 25% of the weekly health reports [12, 21]. All data were processed using Excel (version 16.74) and SPSS (version 27). Prevalence was calculated by dividing the number of dancers with a health problem by the number of all dancers and expressed as percentage. Incidence proportion was calculated by dividing the number of health problems by the number of dancers and expressed as mean. Incidence per 1000 dancer-days was calculated by dividing the number of health problems by the number of days documented (i.e. number of returned weekly report forms multiplied by 7 days) multiplied by 1000. Statistical methods applied were descriptives, correlation, t- and chi^2^-test. Significance was accepted at *p* < 0.05. Results were reported as number with percentages or mean with standard deviation.

## Results

### Study Group

Ninety-six professional dancers from three German ballet companies agreed to participate and filled in the baseline questionnaire. Of these, 57 dancers (59.4%) filled in at least 25% of the weekly health reports during the 44 weeks of the season. The 33 (57.9%) female and 24 (42.1%) male dancers were on average 28.1 years old (sd = 5.2, range 19–37 years) without difference between genders. All dancers had a professional dance education and had worked for a professional company for an average of nine years. All were full time employed (40 h/week) at their current company. The companies had on average one to two performances per week in the 2021/22 season. Most dancers (*n* = 37, 64.9%) classified themselves as corps de ballet/semi-soloist/coryphées, 15 (26.3%) as soloist, one (1.8%) as eleve, three (5.4%) in more than one category, and one did not answer this question.

### Response to the Weekly Health Reports

**Fig. 1:**
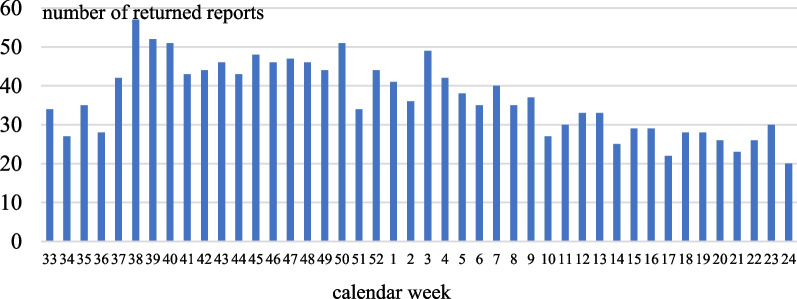
Returned health reports in each calendar week of the season 2021/22

The 57 dancers filled in a total of 1627 weekly reports during the 44 weeks of the 2021/22 season. On average every dancer returned 28.5 reports which is a response rate of 64.9%. The response was similar between male and female dancers (Table 1) and rate dropped towards the end of the season (Fig. 1).

**Table 1:** Number of received weekly health reports, response rate and number of reported health problems by female and male dancers during one season

	Female dancers (*n* = 33)	Male dancers (*n* = 24)
*Health reports*		
Number of weekly health reports	988	639
Average number of weekly health reports per dancer	29.9	26.6
Response rate	68.0%	60.5%
Reports with at least one health problem (%)	614 (62.1%)	406 (63.5%)
*Reported health problems*		
Injuries	148 (69.2%)	86 (81.1%)
Illnesses	37 (17.3%)	17 (16.0%)
Mental health problems	29 (13.6%)	3 (2.8%)

### Severity of Musculoskeletal Pain, all Health Problems and Impaired Ability to Dance

**Fig. 2:**
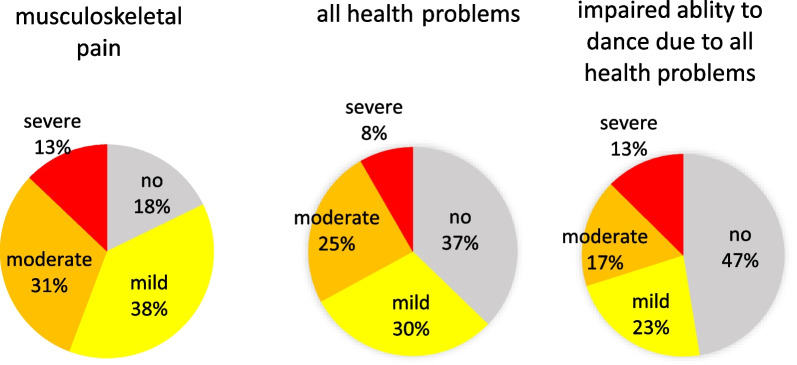
Percentage of weekly health reports with differed severity of musculoskeletal pain, all health problems and impaired ability to dance at full potential due to all health problems in the last 7 days on numerical rating scales (NRS) ranging from 0 to 10 (no: NRS = 0; mild: NRS = 1 to 3; moderate: NRS = 4 to 6; severe: NRS = 7 to 10)

The dancers rated the severity of musculoskeletal pain on average 3.3 (sd = 2.5), of all health problems on average 2.5 (sd = 2.5) and of impaired ability to dance at their full potential due to their health problems on average 2.4 (sd = 3.1) without gender difference. The proportion of health reports with different severity of musculoskeletal pain, all health problems and impaired ability to dance due to health problems are presented in Fig. 2. The severity of musculoskeletal pain correlated moderately with the severity of all health problems (*r* = 0.54, *p* < 0.001) and less with the rating of impaired ability to dance at full potential (*r* = 0.37, *p* < 0.001), while the correlation of the latter two variables was high (*r* = 0.79, *p* < 0.001).

### Frequency, Type and Consequences of Health Problems

The dancers stated at least one health problem in 1020 (62.7%) of the 1627 weekly reports. Thus, on average, a dancer reported a health problem more often than in every second week. In most weekly reports one health problem (*n* = 795, 61.4%) was described, in 186 reports two health problems, in 28 reports three health problems, and in 11 reports four health problems. In total, 1295 reports of a health problem were received of which a quarter (*n* = 320, 24.7%) were newly reported health problems and three quarters (*n* = 975, 75.3%) were follow-up reports of a health problem reported previously during this season. Thus, every dancer reported on average 5.6 health problems during the season and each health problem was reported in, on average, four weeks. The health problems resulted in at least 3313 days when the dancer was not able to dance at full potential, including 1114 days when the dancer was completely unable to dance, this is equivalent to 29.1% resp. 9.8% of the 11,389 days documented in the weekly reports.

Almost three quarters of the health problems were injuries (*n* = 234, 73.1%), 54 (16.9%) illnesses and 32 (10.0%) mental health problems. The results for female and male dancers are presented in Table 1.

### Prevalence, Incidence Proportion and Characteristics of Injuries

Almost all dancers (*n* = 55, 96.5%) reported an injury and two thirds (*n* = 38, 66.7%) at least one time-loss injury during the season. In total, 234 injuries were reported resulting in an injury incidence proportion of 4.1 (95%CI 3.6–4.6). The prevalence and incidence proportion were similar in male and female dancers (Table 2).

**Table 2:** Prevalence, incidence and characteristics of injuries reported by female and male dancers during one season

Injuries	Female dancers (*n* = 33)	Male dancers (*n* = 24)
Prevalence of injury	97.0%	95.8%
Prevalence of time-loss injury	63.6%	70.8%
Incidence proportion	4.5 (95%CI 3.8–5.2)	3.6 (95%CI 2.8–4.4)
Incidence per 1000 dancer-days	21.4 (95%CI 18.0–24.9)	19.2 (95%CI 15.1–23.3)
Injured body part	*n* = 148	*n* = 86
Neck/cervical spine	11 (7.4%)	6 (7.0%)
Upper back/thoracic spine	5 (3.4%)	7 (8.1%)
Lower back/lumbo-sacral spine/buttock	15 (10.1%)	8 (9.3%)
Chest/ribs /abdomen	3 (2.0%)	2 (2.3%)
Shoulder	8 (5.4%)	2 (2.3%)
Arm/hand	1 (0.7%)	6 (7.0%)
Hip	17 (11.5%)	5 (5.8%)
Groin	0	1 (1.2%)
Thigh	7 (4.7%)	4 (4.7%)
Knee	8 (5.4%)	10 (11.6%)
Lower leg	18 (12.2%)	12 (14.0%)
Achilles tendon	5 (3.4%)	2 (2.3%)
Ankle	30 (20.3%)	8 (9.3%)
Foot, toe	13 (8.8%)	10 (11.6%)
Others, multiple	5 (3.4%)	3 (3.5%)
New, recurrent or pre-existing	*n* = 148	*n* = 86
New (i.e. the dancer never had this before)	84 (56.8%)	54 (62.8%)
Recurrent after full recovery	14 (9.5%)	13 (15.1%)
Worsening of a not fully recovered problem or a chronic problem	50 (33.8%)	19 (22.1%)
Onset of injury	*n* = 145*	*n* = 86
Suddenly (i.e. in a single instant or over several seconds)	40 (27.6%)	17 (19.8%)
Gradually	78 (53.8%)	37 (43.0%)
Mixed	27 (18.6%)	32 (37.2%)
Activity at onset of injury	*n* = 145*	*n* = 86
Performance	8 (5.4%)	7 (8.1%)
Rehearsal—run-through	28 (18.9%)	25 (29.1%)
Rehearsal—learning new material/sequences of movements	25 (16.9%)	15 (17.4%)
Rehearsal—improvisation	2 (1.4%)	1 (1.2%)
Training/ballet class	37 (25.0%)	24 (27.9%)
Warm-up or cool-down for dance	13 (8.8%)	0
Supplemental training (e.g. conditioning/gym work, Pilates, Yoga)	5 (3.4%)	4 (4.7%)
Others	10 (6.8%)	2 (2.3%)
Activity cannot be specified due to gradual onset	17 (11.5%)	8 (9.3%)

* Classification missing for 3 injuries

The injuries affected most frequently the ankle (*n* = 38, 16.2%), lower leg (12.8%), foot (*n* = 23, 9.8%) and lower back (*n* = 23, 9.8%). Female dancers had more hip and ankle problems while male dancers had more complaints at the knee and foot. The dancers classified most injuries as new (*n* = 138, 59.0%), 50 (21.4%) as chronic, 27 (11.5%) as recurrent after full recovery and return-to-dance, and 19 (8.1%) as worsening of a not fully recovered problem, without significant differences between genders. About half of the injuries (*n* = 115, 49.1%) started gradually, a quarter suddenly (*n* = 57, 24.4%) and another quarter mixed (*n* = 59, 25.2%). The onset of injury differed significantly between male and female dancers (chi^2^ = 17.7, *p* = 0.008, see Table 2). Most injuries occurred during rehearsals (*n* = 96, 41.6%) and a quarter during training (*n* = 61, 26.1%). Injuries during performances, warm-up or supplemental training were rare. For results of female and male dancers see Table 2.

Asked for the perceived reason or cause of their injury, the dancers selected “too much workload” (*n* = 77, 35.3%) most frequently, followed by “tiredness/exhaustion” (*n* = 49, 22.4%) and “stress/overload/insufficient regeneration” (*n* = 47, 21.6%). “Demanding or challenging movements (*n* = 38, 17.4%), “new dance style/repertoire” (*n* = 33, 15.1%), “daily schedule/time of training or rehearsal” (*n* = 33, 15.1%), “previous injury” (*n* = 31, 14.2%) and “insufficient or inappropriate training” (*n* = 25, 11.5%) were less frequent. “Insufficient warm-up” (*n* = 16, 7.3%), “others” (*n* = 18, 8.3%), “dance floor” (*n* = 9, 4.1%), “indoor temperature, ventilation, air condition” (*n* = 8, 3.7%), “other dancer” (*n* = 5, 2.3%), “concentration problems” (*n* = 5, 2.3%), and “scenery/costume/backstage conditions” (*n* = 3, 1.4%) were rare reasons or causes of injury. The dancers did not know a reason for 26 injuries and did not answer the question for 16 injuries. For half of the injuries more than one answer choice was selected (*n* = 109). “Too much workload” (*n* = 18) was also the most frequent single reason reported.

A total of 851 follow-up reports on these injuries were received in the subsequent weeks. Thus, on average each injury was reported in four to five weeks. In 62.6% of the 1085 weeks when the dancers reported an injury, they consulted a health professional because of this injury, mainly a physiotherapist (*n* = 627, 58.7%) and/or a physician (*n* = 128, 12.0%).

### Prevalence, Incidence Proportion and Characteristics of Illnesses

Almost two thirds (*n* = 37, 64.9%) of the dancers reported an illness and about half (*n* = 30, 52.6%) at least one time-loss illness during the season. In total, 54 illnesses were reported, resulting in an incidence proportion of 0.95 (95%CI 0.7–1.2). The prevalence and the incidence proportion of illnesses were similar in female than in male dancers (Table 3).

**Table 3:** Prevalence, incidence and characteristics of illnesses reported by female and male dancers during one season

Illness	Female dancers (*n* = 33)	Male dancers (*n* = 24)
Prevalence of illnesses	75.8%	50.0%
Prevalence of time-loss illness	57.6%	45.8%
Incidence proportion of illnesses	0.8 (95%CI 0.5–1.1)	0.5 (95%CI 0.2–0.8)
Incidence of illnesses per 1000 dancer-days	5.3 (95%CI 3.6–7.0)	3.8 (95%CI 2.0–5.6)
Kind of illness or complaints^#^	*N* = 37	*N* = 17
Cold, influenza/”flu”, angina or similar	19 (51.4%)	11 (64.7%)
Gastro-intestinal problem, e.g. diarrhoea	3 (8.1%)	2 (11.8%)
Menstrual pain/cramps	2 (5.4%)	0
Allergy, e.g. hay fever	0	0
Headache, migraine	7 (18.9%)	0
Other	8 (21.6%)	6 (35.3%)
*New, worsening or chronic*		
New	35 (94.6%)	16 (94.1%)
Worsening or chronic	2 (5.4%)	1 (5.9%)

# Kind of illness or complaints sum up to more than 54 (100%), since multiple answers were selected for 4 (7.0%) illnesses

Most illnesses (*n* = 30, 55.5%) were related to the respiratory system such as cold, flu, angina or similar. The illness listed most frequently under “others” was COVID-19 (*n* = 9). Almost all illnesses were newly incurred during the season. For results for female and male dancers see Table 3.

In addition, 53 follow-up reports of these illnesses were received in subsequent weeks. Thus, each illness was present on average in at least two weeks. In 29.0% of the 107 weeks when dancers reported an illness, they consulted a physician (*n* = 16, 15.0%), a physiotherapist (*n* = 4, 3.7%), and/or another health professional (*n* = 18, 16.8%) because of this illness.

### Prevalence, Incidence Proportion and Characteristics of Mental Health Problems

More than a quarter (*n* = 16; 28.1%) of the dancers reported a mental health problem and five (9.4%) dancers a time-loss mental health problem in at least one week of the season. In total, 32 mental health problems were reported, resulting in an incidence proportion of 0.56 (95%CI 0.4–0.8). Both the prevalence and the incidence proportion of mental health problems were significantly higher in female than in male dancers (Table 4).

**Table 4:** Prevalence, incidence and characteristics of mental health problems reported by female and male dancers during one season

Mental health problems	Female dancers (*n* = 33)	Male dancers (*n* = 24)
Prevalence of mental health problem	42.4%	8.3%
Prevalence of time-loss mental health problem	12.1%	4.2%
Incidence proportion	0.9 (95%CI 0.6–1.2)	0.1 (95%CI − 0.01–0.2)
Incidence per 1000 dancer-days	4.2 (95%CI 2.7–5.7)	0.7 (95%CI − 0.1–1.4)
Kind of mental health problems^#^	*n* = 29	*n* = 3
Depression, low mood	16 (55.2%)	2 (66.7%)
General anxiety	16 (55.2%)	0
Burnout, chronic fatigue	9 (31.0%)	0
Eating disorders	6 (20.7%)	0
Performance anxiety/stage fright	2 (6.9%)	0
Sleeping problems	1 (3.4%)	1 (33.3%)
Other	5 (17.2%)	1 (33.3%)
*New, worsening or chronic*		
New	12 (41.4%)	3 (100%)
Worsening or chronic	17 (58.6%)	0

# Kind of mental health problems sum up to more than 32 (100%), since multiple answers were selected for 18 (56.3%) mental health problems.

For more than half of the mental health problems multiple answers were selected (*n* = 18, 56.3%), in 14 cases just one answer. Depression, low mood (*n* = 18, 54.1%) and general anxiety (*n* = 16, 50.0%) were the most frequently reported mental health problems. The comments listed under “others” concerned most frequently high workload and feeling overwhelmed, overloaded or stressed.

In addition, 71 follow-up reports of these mental health problems were received in subsequent weeks. Thus, on average, the mental health problems were present in at least three weeks. In 37.9% of the weeks when the dancers reported a mental health problem, they consulted a physician (*n* = 7, 6.8%), a physiotherapist (*n* = 18, 17.5%), or another health professional (*n* = 15, 14.6%) because of this problem.

## Discussion

This study used weekly self-reports to determine the burden of health problems in 57 male and female professional ballet dancers of three companies during one season. In total, 1627 health reports covering 11,389 dancer-days were received. The dancers reported musculoskeletal pain of different degrees in 82% of the weeks and felt that their ability to dance at their full potential was affected in about every second week (52.6%). The correlation of severity of musculoskeletal pain and impairment to dance was low (*r* = 0.37), as reported previously [17, 22]. Almost all dancers reported a health problem during the season, a similar prevalence has been reported previously for dance students [15].

### Musculoskeletal injuries

In the present study, the season prevalence of injuries was 96.5% and the incidence proportion was 4.1 without significant difference between genders. This incidence proportion is similar to the results for professional dancers reported in two meta-analyses [1, 2], and both prevalence and incidence proportion were just slightly higher than reported for medical attention injuries of the dancers from the Royal Opera House in London [5]. The prevalence of time-loss injuries of female (63.6%) and male (70.8%) dancers in our study were within the range of the latter study [5]. Also, the characteristics of injuries were similar to previous studies: the ankle, foot and lower back were most frequently affected [2, 5, 23, 24], the majority of injuries had a gradual or mixed onset and thus, were most likely overuse injuries [1, 5, 23], about 60% of the injuries were the first episode [5], and most injuries were incurred during rehearsals [5]. However, while at the Royal Opera House about 20% of injuries were incurred during performances [5], in our study it were only 6.4% whereas more injuries were sustained during training (26% vs 15%).

In the present study, the prevalence and incidence of injury was similar in female and male dancers, but gender differences were observed in the location of injury which is in accordance with previous studies on professional ballet dancers [23, 25]. The ankle was most frequently injured in female dancers [25], while it was the lower leg in the male dancer [23].

About 20 years ago, Byhring & Bo [24] reported that dancers believed the risk of injuries was related to training, organizational and environmental factors. In the present study dancers regarded “too much workload” and “tiredness/exhaustion” and “stress/overload/insufficient regeneration” as the main reason or cause of injury. This result supports the observation of Bolling et al. [17] that dancers perceived the imbalance between workload and their capacity to deal with load as the main cause of injuries. This again leads to important implications for injury prevention for which either the workload for dancers needs to be reduced or dancers should be better prepared for the demands of their training, rehearsals and performances and especially the routine training should be modified to minimize the risk of injury. It has been reported that dancers often demonstrate low levels of aerobic [26] or cardiorespiratory fitness [27] even though a strong aerobic foundation is necessary to meet the required workload [26]. Further, it has been shown that supplementary low-intensity aerobic training can improve aerobic capacity without affecting the psychomotor performance of professional female ballet dancers [28]. Injury prevention programmes should include specific exercises to protect the most frequent injuries in body parts, such as ankle, foot, lower back, hip and knee [2, 5, 23, 24]. Recent studies on injury prevention programmes in (pre-)professional dancers showed promising results [29–31].

### Illnesses

In our study the incidence proportion of illness was about one, most illnesses affected the upper respiratory tract, and more than half (52.6%) of the dancers reported a time-loss illness during the season. The prevalence and the incidence proportion were higher in female than in male dancers. The very few studies that investigated illnesses of dancers support the finding that illness seems to be a substantial health problem in dancers. Van Winden et al. [15] stated that 37.5% of the reported health problems of dance students were illnesses. Another study by Jeffries et al. [18] registered 134 illness episodes of upper respiratory tract infections during one year in 16 professional contemporary dancers. Upper respiratory tract infections are the most common illnesses in both athletic and nonathletic populations [32]. A relation between training load and illness has been discussed in athletes and seems to be different in recreational and sub-elite athletes (S-curves) and elite athletes (J-curve) [33]. General guidelines to prevent illness in athletes include behavioural, lifestyle, nutritional and medical interventions [33].

### Mental Health Problems

In the present study, about 10% of the reported health problems concerned mental health which is similar to dance students [15]. The incidence proportion in our study was slightly higher (56%) than in dance students (45% [19]) which might be explained by the frequency of data collection (weekly vs monthly). In our study 28% of the professional dancers reported a mental health problem during the season, while 29% of the students reported a mental health issue as their most severe health problem [19]. Thus, the season prevalence of mental health problems seems to be higher in students than in professional dancers. While in our study the prevalence was substantially higher in female than in male dancers, no gender difference was found in dance students [19]. A higher prevalence of mental health symptoms in women than in men has been reported for general populations [34, 35], elite athletes [36, 37] and in professional dancers [38]. Similar to the general population, the most frequent symptoms in the present study were “depression, low mood” and “generalized anxiety”. In dance students they were “general anxiety” and “stress due to external factors” [15]. Potential reasons for mental health problems of dancers (e.g. body image pressure, chronic injury, career uncertainty, training load) have been discussed in the literature [38–40]. However, few preventive interventions have been developed and evaluated [41].

### Limitations

Since participation in the project was voluntary not all dancers employed at the three companies could be included in the study. Of the 96 dancers who participated in the baseline examination 57 answered at least 25% of the weekly health reports during the season. Thus, the results might not be representative for all dancers of the three companies. But almost all previous studies focus on just one company while dancers from three companies were included in the present study. The response rate to the weekly health reports was 64.9% and thus, the prevalence and incidence proportion of mild health problems might be slightly under-estimated. However, the prevalence, incidence proportion and characteristics of injuries were similar to the results of two meta-analyses [1, 2] and previous studies [5, 23, 24]. It can also not be excluded that some dancers did not report all their medical problems because they fear potential consequences [14, 42], although confidentiality was assured. But this can also apply to reports of in-house medical staff when dancers want to conceal their medical problems [3]. In this context, it is important to consider that in Germany the contracts of almost all dancers are limited to one year only. Further, most of the German states theatres and opera houses don´t have in-house medical staff, and thus, using dancer´s self-reports was the only way to collect data on their medical problems. While some information, such as a medical diagnosis, should be based on examination by qualified health care professionals, others, such as severity of pain and/or complaints, can only be provided by the dancer themselves. Therefore, both data collection methods have advantages and disadvantages. Finally, no data on exposure time was collected in the present study and thus, a calculation of injury incidence per 1000 h was not possible. Incidences were provided as incidence proportion and in relation to 1000 dancer-days which is more adequate for illnesses and mental health problems.

## Conclusion

Professional dancers have a high burden of health problems. While almost three quarters of the health problems were injuries, about two thirds of the dancers reported at least one illness and more than a quarter a mental health problem during one season. Therefore, illness and mental health problems should be included in future epidemiological studies. Further, preventive interventions are urgently required to reduce the dancers´ high prevalence of injury. Injury prevention measures should regard the balance of the load capacity of professional dancers and the workload in training, rehearsals and performances.

## Data Availability

Due to confidentiality reasons, no data can be shared.

## Abbreviations

95%CI: 95% confidence interval
NRS: Numeric rating scale
PAHM: Performing artist and Athlete Health Monitor
SD: Standard deviation
SPSS: Statistical package für social sciences
